# Growth stimulation of two legumes (*Vicia faba* and *Pisum sativum*) using phosphate-solubilizing bacteria inoculation

**DOI:** 10.3389/fmicb.2023.1212702

**Published:** 2023-08-14

**Authors:** Walid Janati, Karima Mikou, Lahsen El Ghadraoui, Faouzi Errachidi

**Affiliations:** Functional Ecology and Environment Engineering Laboratory, Faculty of Science and Technology, Sidi Mohamed Ben Abdellah University, Fez, Morocco

**Keywords:** alkaline phosphatase, broad bean and pea, PSBs inoculation, plant growth promotion, rock phosphate

## Abstract

The application of chemical fertilizers for plant growth and protection is one of the reasons for the environment and ecosystem destruction, thus, sustainable agriculture is gaining popularity in research and among farming communities. Although most soils are high in total phosphorus (P), a large portion is unavailable to plants and regarded as a growth-limiting factor. P-solubilizing bacteria (PSB) exploitation is a newly developed bio-solution for enhancing rhizosphere P availability; however, the effect of these bacteria on soil quality and the different phases of plant growth remains unknown. This study aims to evaluate the impact of five strains of PSB, isolated from legume rhizosphere, on the growth of two plants (*Vicia faba* and *Pisum sativum*) and certain soil properties. The efficient strains of PSB used are characterized by the P-solubilization, the ACC deaminase activity, the fixation of N, and the IAA, HCN, and siderophores production. The activity of these bacteria is tested *in vitro* and *in vivo* under controlled conditions on the growth of the two plants supplemented with the rock P (RP). According to our findings, all PSBs strains outperformed the control in terms of enhancing the growth of the tested legumes with a percentage ranging from 77.78 to 88.88%, respectively. The results showed that all treatments significantly improved plant parameters like nitrogen- (N) and P-content in the plants (67.50, 23.11%), respectively. Also, an increase in the fresh and dry weights of above- (41.17, 38.57%) and below-ground biomasses (56.6, 42.28%), respectively. Compared to the control, this leads to an increase of 72% in root length, 40.91% in plant dry weight, and 40.07% in fresh weight. Rhizospheric soil in PSBs treatments displayed high levels of N, P, and organic matter. All treatments were found to have significantly higher levels of alkaline phosphatase, basal soil respiration, and β-glucosidase activity than the control. It is concluded that multi-traits PSB can be an alternative for utilizing chemical fertilizers to enhance soil quality and plant growth. Despite the potency of PSBs, its use as a source for the development of sustainable agriculture implies focusing on crop species and adaptation, stress tolerance and climate resilience.

## Introduction

More food is needed to feed the world’s growing population, but agricultural yields are decreasing due to abiotic stressors such as nutrient shortage, drought, salinity, and temperature, which are the main obstacles to sustainable agriculture worldwide (Kapadia et al., 2021). It has become increasingly important in recent years to address the food demand without endangering ecosystems. Due to the significant losses and escape to the environment as a result of various processes, such as denitrification in the case of nitrogen fertilizers and eutrophication in the case of phosphorus fertilizers, both organic and inorganic fertilizer applications to agricultural fields are frequently criticized (Namli et al., 2017). Along with ecosystem pollution, extensive use of mineral fertilizers is also leading to land degradation. Researchers have paid close attention to these circumstances, which force us to improve soil fertility by implementing eco-friendly and sustainable agriculture, which is possible solely using biofertilizer-based microorganisms. Indeed, using these soil microorganisms for agricultural purposes can improve soil nutrients while reducing farmers’ reliance on inorganic fertilizers (Zaidi et al., 2017).

The most important nutrient for plant growth and development during an adequate rate from the earliest stages of their development is phosphorus (P), which is second only to nitrogen (N). This element is crucial to plant metabolism and multi-functions including developing roots, root anatomy, and root hair density, all of which significantly increase crop yield and plant resistance to various diseases (Khan M. et al., 2022). P is crucial at the cellular level as it is involved in cell division, tissue growth, and nucleic acid structure, all of which regulate protein synthesis, energy transfer, and photosynthesis (Malhotra et al., 2018). Except, P exists in various forms in the soil but a significant portion is insoluble due to fixation and precipitation by soil minerals like aluminium, iron, and calcium, it is absorbed from the soil solution largely as monovalent (H_2_PO_4_^−^) and divalent (HPO_4_^2−^) orthophosphate anions in proportions relative to the soil pH (Ducousso-Détrez et al., 2022). Accordingly, it has been estimated that less than two billion hectares of agricultural land worldwide are affected by low P availability, and P deficiency directly causes a significant reduction (5%–15%) in crop yields (Shenoy and Kalagudi, 2005; Elhaissoufi et al., 2022). According to Alori et al. (2017), the release of various organic acids by microorganisms causes the acidification of microenvironments, which in turn causes P ions to be replaced by cations, a process known as phosphorus solubilization (Kalayu, 2019). For this reason, P-solubilizing microorganisms can convert insoluble P into plants’ P-available forms. However, given their range of activities, P-solubilizing bacteria (PSB) are the most significant soil microbes for P-solubilization. Along with increasing P availability for the plants, several PSBs likewise contribute to the production of siderophore, indole acetic acid (IAA), and gibberellin, as well as antibiotics, secondary metabolites, and 1-aminocyclopropane-1carboxylate (ACC) deaminase enzyme (Lee et al., 2019; Mitra et al., 2020; Zhao et al., 2021; Song et al., 2022). In addition, PSBs are also believed to be effective biocontrol agents for soil-borne pathogens (Yadav and Yadav, 2022). Therefore, PSB utilization represents a promoting issue to increase plant growth and P availability in soil. When compared to the control, Namli et al. (2017) found that PSB inoculation treatments significantly increased above and below-ground plant biomasses (fresh and dry weight), plant N, and P content. Likewise, Biswas et al. (2018) noted that the sterilized seeds of mung beans (*Vigna radiata*) displayed a greater germination rate and higher growth under PSBs inoculation. Additionally, according to Song et al. (2022), PSBs from intercropped soybean rhizosphere soil may secrete organic acids that increase the solubilization of unavailable P, enhance soil ACP activity and P availability, and produce IAA and siderophores that aid in the germination and growth of maize seeds. On their part, Valetti et al. (2018) presented that all the PSB strains used in their experiment were able to promote plant growth under greenhouse conditions and also increase rapeseed yield (from 21 to 44%) in field trials.

Since PSBs can play a key role in enhancing the host plant’s ability to withstand stressful conditions such as nutrient deficiency, we addressed the question: are the performance and plant growth promotion mechanisms of rhizobacteria associated with the crop species? Considering the importance of using PSBs, we hypothesize that legume seed inoculation could enhance soil fertility and improve crop yields. In addition to their important role in the integrated management of soil fertility, their ability to fix atmospheric N_2_ in association with rhizobia may result in improved soil fertility and reduced reliance on N fertilizers in agricultural systems (Vanlauwe et al., 2019); legumes are an essential source of vegetable protein for human and animal nutrition (Stagnari et al., 2017). This is consistent with the study’s goal and justifies the choice to work on the two species of this family to examine the process by which five P-solubilizing rhizobacteria performed. Specifically, we assess the effects of PSBs applications on some soil chemical properties such as pH, electrical conductivity (EC), organic matter, N, and P in addition to the biological characteristics like rhizosphere’s basal soil respiration, alkaline phosphatase activity, and β-glucosidase activity. Furthermore, different growth parameters consistently germination rate, nutrient content, plant biomass, shoot length, root depth, and yield of Broad Bean and Pea plants. The study’s findings may offer a practical method for agronomically enhancing microbial inoculants to increase soil P mobilization for plant growth in intensive production or agroecological applications. This work contributes to Morocco’s national strategy (GREEN GENERATION 2020–2030), which aims to achieve sustainable development and maximise yields. This involves improving soil quality using biological methods.

## Materials and methods

### Phosphate solubilizing bacteria isolation and P activity

Five PSB isolates based on their P-solubilizing capacities (PSC) (low, moderate, and high P-solubilizers) served as inoculants in this study to explore the above- and below-ground physiological responses of rock P (RP)-fertilized Pea and Broad Beans seeds. These isolates were among 51 PSB isolates collected from the soil rhizosphere (strongly adhering to the roots) of legumes at four agricultural production sites in the Fes-Meknes region (Tafrant, Ait Yaakoub, Dayt Al Amira, and Ait Saleh). The National Botanical Research Institute (NBRIP)-agar P growth medium was used for PSBs isolation, with either tricalcium P (TCP, Ca_3_ (PO_4_)_2_, 5 g/L) or RP (5 g/L containing 28.15% of P_2_O_5_) as the sole source of P added with (per litre); glucose: 10 g; MgCl_2_ · 6H_2_O: 5 g; MgSO_4_ · 7H_2_O: 0.25 g; KCl: 0.2 g and (NH_4_)_2_ SO_4_: 0.1 g (Nautiyal, 1999). Colonies were selected according to the development of phosphate solubilization zones and quantitative analysis of P solubilization rates in NBRIP liquid medium supplemented with TCP or RP was performed after seven days of incubation at 28°C. The available P portion was determined by a spectrophotometer at 880 nm using the ascorbic acid colourimetric method. The absorbance was measured using standard solutions of KH_2_PO_4_ with P concentrations ranging from 0.1 to 1 mg.L^−1^ (Janati et al., 2022).

### Bacterial characterization for plant-growth-promoting traits

In addition to P solubilization, isolates were tested for plant growth-promoting (PGP) traits such as N-fixation (ammonium producers), siderophores production, indole acetic acid (IAA) production, 1-aminocyclopropane-1-carboxylic (ACC) deaminase activity, and hydrogen cyanide (HCN) production.

### Analysis of IAA production

According to Gordon and Weber (1951) methods, IAA production by PSBs was determined with some revisions. The strains were inoculated separately into an LB liquid medium containing L-tryptophan (1 L + 0.1 g w/v). The suspension was incubated at 28 ± 2°C for 4 days. The broth was centrifuged at (30,000 xg, 10 min). 0.1 mL of the supernatant was mixed with 0.1 mL of Salkowski’s reagent (4.5 g FeCl_3_ and 587.4 mL of 98% H_2_SO_4_, solution) and kept in the dark for 15 min (Biswas et al., 2018). The absorbance at 530 nm was measured using a standard solution of IAA varying in concentration from 0 to 100 μg.mL^−1^. The content of IAA per unit volume of fermentation broth was calculated according to the standard curve.

### ACC deaminase activity

The isolates’ growth in DF minimal salts medium (Dworkin and Foster, 1958) augmented with 3 mM ACC was examined to determine whether they could use ACC as an N source. The absorbance at 540 nm, which is the-ketobutyrate produced from the enzymatic cleavage of ACC through ACCD as previously discussed by Honma and Shimomura (1978), was used to measure the ACC deaminase activity of the isolates. By comparing a sample’s absorbance at 540 nm to a standard curve of-ketobutyrate with concentrations between 0.1 and 1.0 mM, the amount of- ketobutyrate produced by this reaction is calculated (Chinnadurai et al., 2009).

### Analysis of N-fixation

In an N-free Ashby medium made up of the following components: agar 15 g, mannitol 15 g, K_2_HPO_4_ 0.4 g, CaCl_2_ · 2H_2_O 0.1 g, NaCl 0.2 g, MgCl_2_ 0.1 g, FeSO_4_ · 7H_2_O 3.0 mg, NaMoO · 2H_2_O 3.0 mg, free N-fixation has been proven to occur. PSB isolates grown in Ashby medium were regarded as free N-fixer isolates after 7 days at 28 ± 2°C. Isolate’s ability to produce ammonium was verified according to Geetha et al. (2014), 10% peptone water was prepared, autoclaved, and inoculated with selected strains. After 3 days of incubation, 0.5 mL Nessler’s reagent was added to 1 mL supernatant. A positive test result was indicated by the development of a brown to yellow colour; the intensity of the colour revealed how much ammonia the isolates had produced.

### HCN production

Hydrocyanic acid (HCN) production was quantified following Bakker and Schippers (1987). On tryptone soya agar plates that had been amended with 0.44% glycine, a few strains were streaked. The filter paper was placed on the agar plate, dipped in a solution of 2% sodium carbonate and 0.5 mL picric acid, sealed with para-film, and incubated for 72 h at 37 ± 2°C. According to the test, the isolates produce HCN because the filter paper’s colour changes from yellow to reddish-brown (Lagzian et al., 2013).

### Analysis of siderophores production

All of the tested strains were inoculated into mannitol salt agar (MSA) liquid medium and shaken for two days at a temperature of 28 ± 2°C at 180 rpm in the incubator. The fermentation broth underwent a 6,800 xg, 10 min centrifugation. Using the chrome azurol sulfonate (CAS) assay, the isolates’ production of siderophores was quantitatively assessed (Shin et al., 2001). A solution containing 10 mL of FeCl_3_ · 6H_2_O solution was added to a solution containing 60.5 mg CAS in 50 mL of deionized water. Additionally, to make CAS’ final volume of 100 mL, 72.9 mg of HDTMA (hexadecyltrimethylammonium bromide), dissolved in 40 mL of deionized water, was added. After thoroughly combining 4 mL of the CAS solution with 4 mL of the culture supernatant, the mixture was incubated for 1 h. At 630 nm, the absorbance of each sample was determined. By measuring the percentage of siderophores units produced by various bacterial strains, siderophores production was assessed (Eq. 1).


(1) SUs=[(Ar−As)/Ar]×100

Ar: absorbance of the reference (CAS reagent) and As: absorbance of the sample.

### Plant and soil used

The effect of bacterial strains on germination and plant growth is tested on two legume crops, Feve-Aguadulce Broad Bean (*Vicia faba*) and PP-Onward Pea (*Pisum sativum*). The seeds were obtained from the agriculture plant section of A.PHY.SEM Company. The soil used in this study was collected from a field on the Science and Techniques Faculty of Fez’s experimental farm. Standard procedures were followed to determine the physical and chemical characteristics of the soil before the experiment began (Pavan et al., 1992). Before and after the culture, the soil’s N, P, and K contents are measured.

### Controlled seed germination experiment

For seedling bioassay, five bacterial isolates with different PSC were grown in TSA agar medium (24 h at 28 ± 2°C). Broad Bean (*Vicia faba*) and Pea (*Pisum sativum*) seeds were surface sterilized with sodium hypochlorite (2%) and washed five times with distilled sterile water. Sterilized seeds were inoculated with bacteria using a suspension containing 10^9^ bacteria.mL^−1^, separately. The seeds are placed in Petri dishes containing sterilized filter paper and incubated for 1 week at 25°C. Seeds are considered to be germinated when the radicle breaks through the seminal envelope. Germination parameters such as germination energy, seed water content, and average germination time were measured during the germination week. After 7 days post-inoculation, the germination percentage was registered. The root length of each seedling was measured seven days after incubation and compared to the non-inoculated seeds.

### Biological assay

After 24 h of incubation, we carried out biological tests using kinetics. During the 6 days of germination, we condemned two seeds (for the two studied legumes, separately) each day for the determination of soluble proteins, protease activity and starch content. To this end, for each sample (two Pea seeds and two Broad Bean seeds), we removed the seed coats and embryos (future seedlings) and kept the cotyledons to carry out the biological tests mentioned above. Physiologically speaking, the main proteins in legumes are reserve proteins, whose role is to be a source of nitrogen, carbon, sulphur and amino acids for the embryo’s future needs. We carried out kinetics to see the effect of PSB inoculated into the seeds, which can improve the concentration of soluble proteins, which is one of the essential characteristics for efficient germination. We then analysed protease activity to validate the positive correlation with soluble protein. Starch is also required to make simpler sugars such as maltose and glucose after hydrolysis by amylase, which is necessary to maintain germination and seedling growth. For this purpose, a kinetic analysis of the starch concentration during the 6 days of germination was required to validate the positive effect of PSBs during the germination of the two leguminous plants tested. So we noticed the induction of proteolytic and aminolytic activities.

### Determination of soluble proteins

For soluble proteins determination, 1 g of fresh material seeds were homogenized in an ice-cold mortar supplemented with 2 mL of phosphate buffer solution, the homogenate is centrifuged (30,000 xg, 10 min at 2°C), and 1 mL of the supernatant is mixed with 2 mL Bradford reagent before incubating in the dark for 15 min. The protein concentration is determined using a reference to a standard solution of bovine serum albumin (BSA) of known concentration. The absorbance was measured at 595 nm (Karimi et al., 2022).

### Determination of protease activity

Protease activity was determined according to Anson (1938) with few modifications. 1 g of fresh material seeds were homogenized in an ice-cold mortar supplemented with 2 mL of phosphate buffer solution, the homogenate is centrifuged (30,000 xg, 10 min at 2°C), and the supernatant (enzyme extract) is recovered. One mL of 2% casein is combined with 250 μL of enzyme extract, and the mixture is then incubated at 37°C for 30 min. The reaction was stopped by adding 500 μL of 10% trichloroacetic acid (TCA). The substrate was treated as previously mentioned for the control after being precipitated with 500 μL 10% TCA before being added to the enzyme solution. After centrifugation, 300 μL supernatant was added to the 2.5 mL cu-alkaline solution and kept for 15 min. The mixture was then thoroughly combined with 250 μL of folin ciocalteau reagent (FCR), which had been diluted two times. Using a spectrophotometer, the absorbance was measured at 660 nm. The difference in absorbance between the test sample and the control sample was used to measure the protease activity.

### Determination of starch content

Starch content was determined according to Allfrey and Northcote (1977) as cited by Dkhil and Denden (2010). Seeds were homogenized in an ice-cold mortar (1 g fresh material) and pestled in a volume of 10 mL 80% (v/v) ethanol. The homogenates were centrifuged (30,000 xg, 10 min at 2°C) and then perchloric acid (HClO_4_; 6 mL, 30%, v/v) was added to solubilize starch from the pellet. The suspension was left at room temperature for 6 h, and starch was detected with an I_2_-KI reagent. Samples of 1 mL starch solution were mixed with 1 mL I_2_-KI reagent, and then were vortexed and left standing at room temperature. The standard curve of 0 to 5 mg.mL^−1^ that was created using soluble starch dissolved in 30% HClO_4_ and detected with the same I_2_-KI reagent was compared to the absorbance at 620 nm.

### Pots trails experiment

Five PSBs strains with different PSCs were selected for the legumes pot experiment (50 mL of bacterial suspension per pot). The experiment included pots control without inoculation and pots with strains inoculation for both Pea and Broad Beans seeds. All pots are supplemented with RP solution (5%) as a P-fertilizer. The experiment was conducted at the experimental greenhouse of the Science and Technology Faculty, Fez, Morocco, from 21 March to 21 May 2022, with an average temperature of 30°C in the daytime and 13°C at night. The experiment used a three-replicate per treatment, fully randomized design. Each pot contained five seeds, which received daily weight-based irrigation equal to 70% of the water-holding capacity. Plants were harvested for root and shoot analyses 60 days after germination.

### Morphological shoots and roots traits measurement

Plants were harvested and divided into shoots and roots 60 days after germination. An analytical balance was used to calculate the fresh weights (FW) and shoots and roots highest (SH, RH). Analysis was done on plant nodulation and biomass. Before being ground to a fine powder for P and N concentration analyses, the shoots and roots of treated plants and control plants were dried in an oven at 70°C for 2 days to determine their dry weights (DW).

### Determination of P and N nutrients acquisition

The rhizosphere growth soil was obtained by carefully separating roots from the adhering soil, which was then sieved (2 mm) before measurements of P and N concentrations. Following ash solubilization in hydrochloric acid (10 N) and finely ground dried samples (0.5 g) that were dried at 100°C for 48 h to determine the total P contents in shoots and roots. The absorbance was measured at 820 nm after the obtained filtrates (1 mL) were added to 5 mL of a reaction mixture containing ammonium molybdate (2.5%) and hydrazine sulfate (0.15%). A total of 100 mg FW of shoots and roots were ground in an extraction solution made up of 5 mL of sodium acetate buffer (0.1 M pH 5.6) and 1 mM dithiothreitol. Homogenates were centrifuged (13,000 xg, 30 min at 4°C) and aliquots of 50 μL were used for quantification of inorganic P (Pi), Shoot and root P contents were measured following the ascorbic acid method (Bargaz et al., 2017). The Kjeldahl method was also applied to the finely ground shoot subsamples (0.5 g) for total N analysis (Magomya et al., 2014). The ratio of plant P content to root dry weight was used to calculate the root P acquisition efficiency (RPAE), which measures the ability of roots to absorb P from the soil (Pan et al., 2008). According to Bargaz et al. (2017), the activity of root acid phosphatase was measured. 100 mg of root FW samples were ground in 5 mL of sodium acetate buffer (0.1 M pH 5.6) containing 1 mM dithiothreitol as part of the extraction process. 50 μL of the supernatant from the centrifugation of homogenates (13,000 xg, 30 min at 4°C) was used to measure the amount of root APase activity. The enzyme activity was measured as the amount hydrolyzing 1 nmol of p-nitrophenyl phosphate (pNPP) per minute per g of root FW.

### Soil analysis after inoculation

Samples of the soil were taken from each pot after the experiment. According to Naseby and Lynch (1997) methods, the chemical characteristics of the rhizosphere soil were determined, including pH-water, electrical conductivity (EC), soil organic matter, N-total and P-available (Fernández et al., 2007), basal soil respiration (BSR), alkaline phosphatase activity (APA), and-Glucosidase activity (GA).

### Statistical analysis

The IBM SPSS Statistics V. 26 software was used to analyze statistical data. The results were shown as means and standard deviations based on three replications. Before performing an analysis of variance (ANOVA), tests for homogeneity of variance and normal distribution were run, and then a Waller-Duncan (W) *post hoc* test was used to determine whether there was a significant difference between the treatment’s mean values at a *p* ≤ 0.05 significance level. Excel Office 2013 is also used for mathematical modelling and regression analysis.

## Results

### Phosphate solubilizing bacteria isolation and screening for PGP traits

All isolates showed separate P-solubilizing halos around bacterial colonies and the P-solubilizing index varied from 1.4 to 3.84. From the 51 PSB isolates presenting different PSCs of TCP, five PSBs were selected WJEF38 (high PSC) with 120.20 mg.L^−1^ solubilized P, 100.81 to 114.93 mg.L^−1^ for WJEF46 and WJEF63 (moderate PSC) and from 75.63 to 78.67 mg.L^−1^ for WJEF15 and WJEF26 (low PSC), respectively (Table 1). Throughout 7 days of incubation, medium acidification with either RP or TCP revealed a sharp decline from an initial value of 7 to 4.195. Additionally, PSBs were determined to be isolates that produced IAA (8.426–91.026 μg.mL^−1^), N_2_-fixers, ammonia producers (24.43–98.37 μg.mL^−1^), ACC deaminase producers (0.27–0.58 nmol α-ketobutyrate mg/protein/h), siderophore-producers (39.46–73.26 Units), and also HCN producers except for WJEF26.

**Table 1:** Phosphate solubilizing bacteria characteristics.

Strains	P-solubilization					PGP traits				
	PSI	P [C] mg.L^−1^		pH		AIA μg.mL^−1^	Siderophores	ACC deaminase	NH_3_ μg.mL^−1^	HCN
		RP	TCP	RP	TCP					
WJEF15	2.46^ab^	72.64^b^	75.63^d^	4.275^d^	4.69^a^	8.43^d^	39.46^c^	0.56^a^	71.2^c^	+
WJEF26	2.30^b^	53.75^c^	78.67^d^	4.675^b^	4.67^a^	−	−	0.27^c^	24.43^e^	−
WJEF38	2.67^a^	77.26^a^	120.20^a^	4.75^a^	4.23^b^	91.03^a^	72.66^a^	0.58^a^	98.37^a^	+
WJEF46	2.27^b^	74.58^ab^	100.81^c^	4.515^c^	4.19^b^	59.78^c^	42.83^b^	0.49^b^	61.89^d^	+
WJEF63	2.33^b^	76.58^a^	114.93^b^	4.78^a^	4.2^b^	71.15^b^	73.26^a^	0.55^a^	85.15^b^	+

Data represent the mean ± of three independent replicates. Values with the same superscript letter were not significantly different according to the Duncan test performed (*p* < 0.05).

### Inoculation effect on the physical and chemical soil properties

The physicochemical properties of the soil were recorded at the beginning of the experiment. Soil data including nitrogen (N), P, and potassium (K) available can be found in Table 2. The experimental soil used in this research was silty clay loam soil with characteristics: humidity of 5%, oxidizable organic matter of 1.08%, and cation exchange capacity (CEC) of 0.19 (dS m^−1^).

**Table 2:** Physicochemical properties of soil used before and after the experiment.

	Organic matter g.Kg^−1^	pH	Total N g.Kg^−1^	Available N mg.Kg^−1^	Total P g.Kg^−1^	Available P mg.Kg^−1^	Total K g.Kg^−1^	Available K mg.Kg^−1^
Before inoculation	12.75	7.88	1.5	18.56	0.79	6.32	9.67	42.44
After inoculation	17.88	5.09	2.4	56.96	1.04	32.14	9.94	46.23

### Effects of PSBs on root growth and seedlings germination *In vitro*

**Figure 1:**
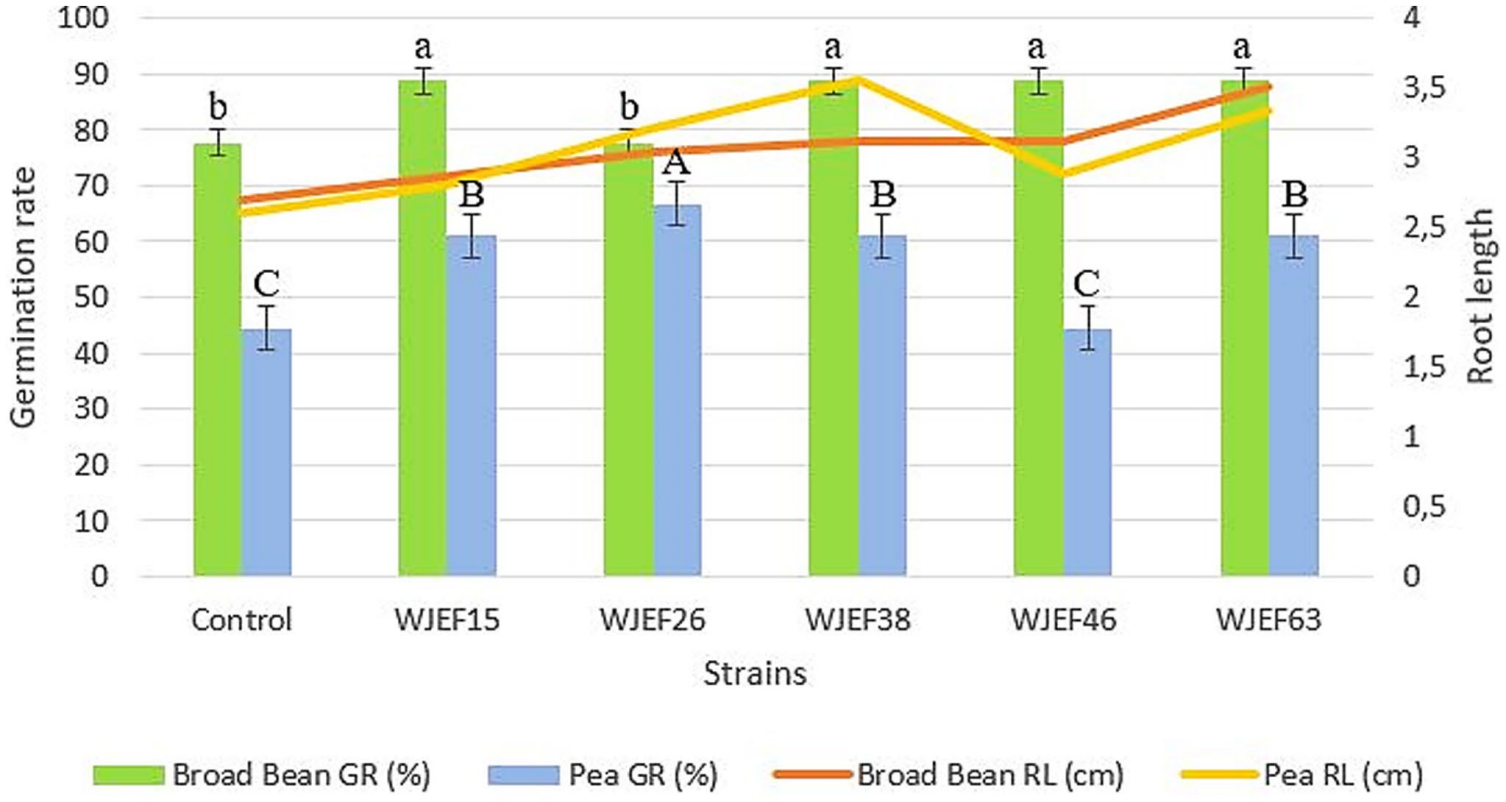
Germination percentage and root length of Pea and Broad Bean seeds inoculated with different PSB isolates. Values with the same superscript letter were not significantly different according to the Duncan test performed (*p* < 0.05).

A petri dish assay was carried out to study the P-solubilizing bacteria effect at the early seedling stage of Pea and Broad Bean. All strains show a significantly positive impact by promoting Pea and Broad Bean seedling growth. The germination percentage and root length compared to the non-inoculated control were increased from 77.78 to 88.88% for Broad Bean seeds and from 44.44 to 66.66% for Pea seeds. The maximum root length results were observed in seeds inoculated, with the WJEF38 strain (3.56 cm) followed by the WJEF63 strain (3.51 cm). We note that these strains also gave the best germination percentage for both plants tested, with an increase of 14.27% for beans and 50% for peas compared to the control. Besides that, the present results showed a significant response regarding germination rate and root length of Broad Bean inoculated seeds better than Pea inoculated seeds (Figure 1). Our results showed a significant relationship between root length and germination percentage of inoculated seeds. Root length increased considerably with increasing germination percentage, which explains a strong positive correlation (*R*^2^ = 0.9239).

### Inoculation effect on the germination parameters

After 7 days, post-inoculation several parameters of germination were evaluated (Table 3). The effectiveness of PSBs on germination energy, average germination time, and water content, confirms the results of germination percentage and root length. This time, as stated in the previous paragraph, the germination parameters of the Broad Bean seeds were also significantly higher than those of the Pea seeds. On the other hand, if we take the water content of the seeds and the average germination time, the increase for the peas is greater than for the Broad Bean. In this case, the efficiency of the strains is more important on Peas than on Broad Bean crops. From the results, we can see that some strains are more efficient than others in certain phenomena. This allows us to say that strains WJEF26, WJEF38, and WJEF63 are the most competent on this level.

**Table 3:** Germination parameters of Pea and Broad Bean seeds inoculated with PSBs over one week.

Strains	Broad Bean			Pea		
	GE (%)	AGT (s/d)	WC (%)	GE (%)	AGT (s/d)	WC (%)
Control	52.77^d^	0.350^d^	32.233^c^	33.33^d^	0.518^b^	44.789^e^
WJEF15	50^d^	0.378^c^	33.592^c^	44.44^b^	0.480^c^	60.186^d^
WJEF26	44.44^e^	0.398^b^	38.764^a^	44.44^b^	0.500^c^	64.133^a^
WJEF38	72.22^b^	0.411^a^	37.739^b^	38.88^c^	0.519^b^	61.556^c^
WJEF46	61.11^c^	0.316^e^	37.876^b^	38.88^c^	0.536^b^	63.390^b^
WJEF63	83.33^a^	0.421^a^	38.337^a^	61.11^a^	0.662^a^	63.333^b^

Data represent the mean± of two independent replicates with five repetitions each. Values with the same superscript letter were not significantly different according to the Duncan test performed (*p* < 0.05). GE, germination energy; AGT, average germination time (seeds/days); WC, water content.

### Protease activity

**Figure 2:**
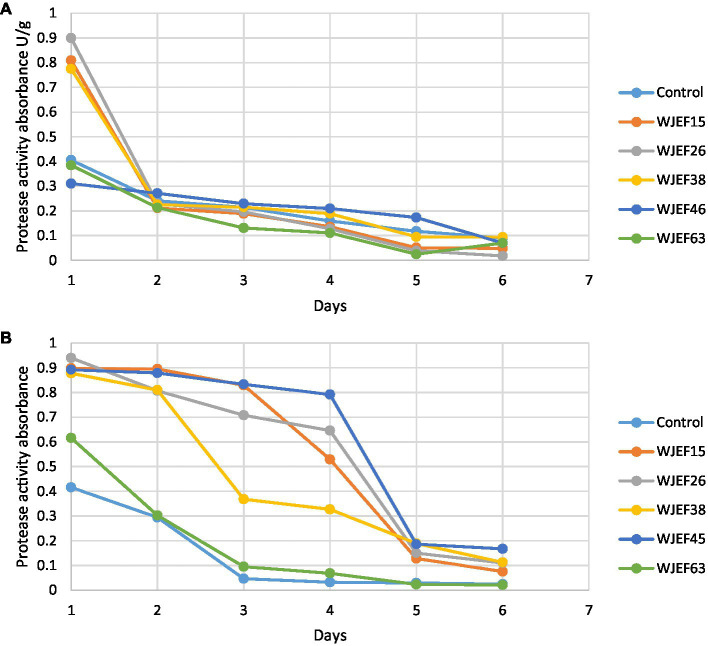
Kinetics of protease activity variation over one week; **(A)** Broad Bean inoculated seeds and **(B)** Pea inoculated seeds.

The protease activity (PA) variation over 1 week is presented in Figure 2. The first 3 days show an important activity. Broad Bean seeds inoculated with strains WJEF26, WJEF15, and WJEF38 have high PA compared to the control. On the other hand, Peas seeds inoculated with WJEF26, WJEF15, WJEF38, and WJEF45 represent the higher PA compared to the control. After 5 days, PA decreases dramatically. Results explain the protein degradation phenomena, which occur between the first and fourth day when germination takes place and thus becomes less intense after the fifth day.

### Protein soluble

**Figure 3:**
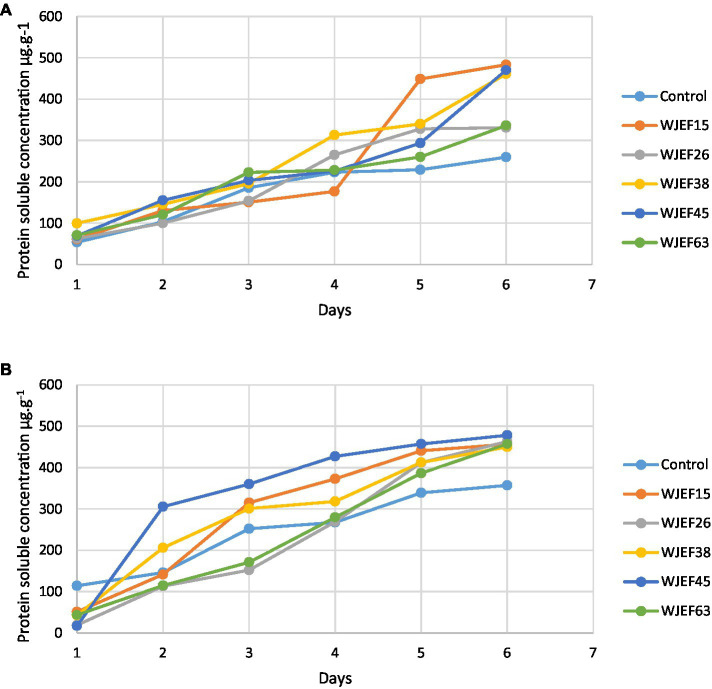
Kinetics of protein soluble concentration over one week; **(A)** Broad Bean inoculated seeds and **(B)** Pea inoculated seeds.

The protein soluble (PS) concentration on the first and third day is almost low for all inoculated seeds including control not exceeding a concentration of 200 μg.g^−1^ FW. From the fourth day, all strains showed high protein content compared to the control (Figure 3). This can be explained by a protease activity that degrades proteins between the first and second day resulting in better germination. Broad Bean seeds inoculated with WJEF15 have a higher PS concentration; however, Peas seeds inoculated with WJEF45 were the most important value.

### Starch content

**Figure 4:**
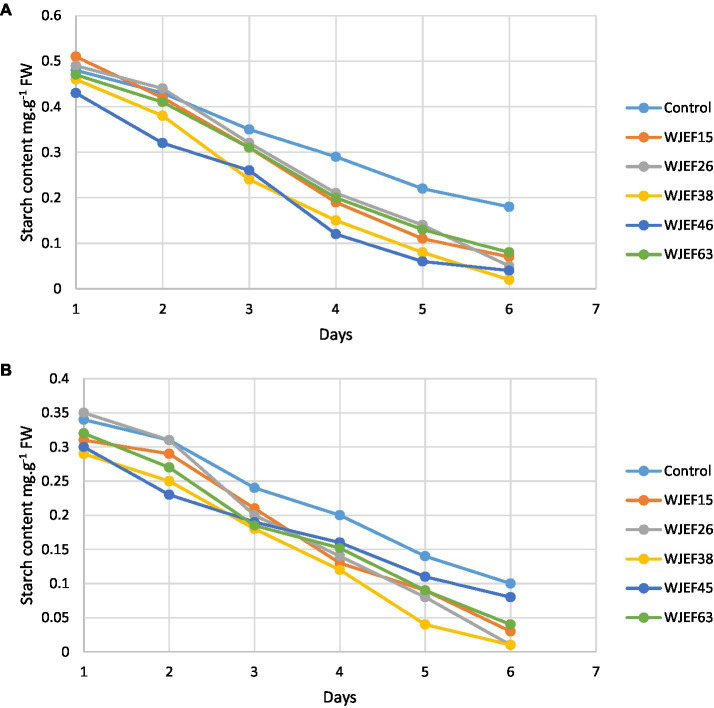
Kinetics of seed’s starch content over one week; **(A)** Broad Bean inoculated seeds and **(B)** Pea inoculated seeds.

Figure 4 shows the effect of PSB isolates on the starch level in germinating seeds Broad Bean and Pea seeds. During germination, the amount of starch decreased clearly and reached 0.02 and 0.01 mg.g^−1^ FW at Broad Bean and Pea seeds, respectively. Starch level decreases more important with inoculated seeds compared to the control. A large decrease of starch was observed with seeds inoculated with WJEF38 and WJEF46 for bot legumes tested.

### Effects of PSBs on plants growth *In vivo*

Phosphate solubilizing isolates were inoculated in Broad Bean and Pea plants to determine their capacity to promote plant growth in greenhouse trials. Eight weeks after inoculation, plants were harvested. All PSB strains showed a significant increase in growth parameters when compared to non-inoculated plants, notably SH, and RL (Table 4). In addition, there has been an increase in SFW and RFW (40 and 55.9%, respectively) in both tested legumes compared to the control. Likewise, SDW and RDW increased by 38.57 and 40.9%, respectively. However, Differences were also noted among PSB isolates, the WJEF38 strain was the significant isolate to increase growth parameters of both tested seeds, reaching values higher than either inoculated or non-inoculated plants. Otherwise, based on the results of SH, RL, SFW, and SDW, we notice that there is an affinity of the PSBs strains with the Broad Bean seeds more than the Pea seeds. On the other hand, we observe that inoculated and non-inoculated plants developed the same number of leaves despite a difference in distance between the nodes. In terms of nodulation, all PSBs strains showed a significant effect when compared to the control; furthermore, we noted a significant variation between PSB strains, whit the WJEF38, WJEF46, and WJEF63 showing the best effect.

**Table 4:** The differences in RP-fertilized broad bean and pea growth in response to PSB isolate inoculation versus RP treatments alone.

Legumes	Strains	SH (cm)	RL (cm)	SFW (g)	RFW (g)	SDW (g)	RDW (g)	Nodulation
Broad Bean	Control	22.90^e^	8.09^d^	2.26^e^	1.63^e^	1.03^e^	0.63^e^	+
	WJEF15	35.68^c^	13.70^c^	2.99^d^	1.87^d^	1.43^d^	0.80^d^	++
	WJEF26	37.69^c^	19.00^b^	3.52^c^	2.25^c^	1.72^c^	1.08^b^	+
	WJEF38	51.01^a^	24.38^a^	4.38^a^	2.62^a^	2.36^a^	1.15^a^	+++
	WJEF46	39.5^b^	20.62^b^	3.92^b^	2.08^b^	1.99^b^	0.84^d^	+++
	WJEF63	31.92^d^	13.00^c^	3.01^d^	1.89^d^	1.13^e^	0.90^c^	++
Pea	Control	20.13^D^	10.78^F^	2.20^E^	1.61^D^	1.08^D^	0.91^D^	+
	WJEF15	25.52^B^	12.47^E^	3.59^C^	2.88^A^	1.59^B^	1.32^B^	+
	WJEF26	22.76^C^	18.87^C^	2.34^D^	2.01^C^	1.11^D^	1.13^C^	++
	WJEF38	36.67^A^	22.66^A^	5.49^A^	2.83^A^	2.67^A^	1.54^A^	+++
	WJEF46	26.33^B^	16.58^D^	3.78^B^	2.37^B^	1.63^B^	1.49^A^	++
	WJEF63	26.02^B^	21.00^B^	3.84^B^	2.79^A^	1.35^C^	1.33^B^	+++

The data are the average of two independent replicates with five iterations each. At *p* < 0.05, values with the same superscript letter did not differ significantly. SH, shoot height; RL, root length; SFW, shoot fresh weight; RFW, root fresh weight; SDW, shoot dry weight; RDW, root fresh weight.

### P and N nutrients acquisition

The PSB inoculation’s contribution was apparent in the response to P fertilization. Seed-inoculated plant parameters were higher than those from the fertilized plant alone. A high positive correlation between plant P content and root acid phosphatase activity in response to PSBs inoculation was observed (Table 5). In our study, higher acid phosphatase activities were found in the roots of seeds inoculated with PSBs, which could accelerate the decomposition of organic and inorganic P and promote plant P uptake. We noted an increase in P availability in the root and an improvement in the plant aerial surface in response to PSB inoculation. With an average increase of 67.50 and 57.24% for Broad Bean and Pea, respectively, compared to non-inoculated plants, shoot N content increased in response to PSBs inoculation. Significantly, higher than the control, a notable increase in RPAE was seen, indicating that PSB strains probably help to improve internal P use efficiency.

**Table 5:** Variations in P content in both root and shoot, root P acquisition efficiency RPAE, shoot total N, and root acid phosphatase activity of plants fertilized with RP and inoculated with PSB strains.

Legumes	Strains	P contents (mg.g^−1^)		Shoot total *N* (mg.g^−1^)	RPAE (mg.g^−1^)	RAPA (nmol∙g^−1^∙min^−1^)
		Shoot	Root			
Broad Bean	Control	2.13^f^	1.08^e^	25.32^e^	3.16^d^	16.52^f^
	WJEF15	3.77^d^	2.21^c^	32.97^d^	7.93^c^	18.26^e^
	WJEF26	3.15^e^	1.89^d^	33.63^c^	8.25^c^	53.56^d^
	WJEF38	4.87^a^	2.45^b^	37.51^a^	10.87^a^	86.89^a^
	WJEF46	4.56^c^	2.73^a^	36.28^b^	10.14^b^	60.28^c^
	WJEF63	4.79^b^	2.66^a^	37.42^a^	10.48^a^	70.64^b^
Pea	Control	1.65^E^	0.76^E^	19.51^E^	2.88^E^	12.62^F^
	WJEF15	3.11^D^	1.34^D^	24.07^D^	4.41^D^	21.73^E^
	WJEF26	3.36^C^	2.27^B^	29.3^C^	5.87^C^	31.02^D^
	WJEF38	4.23^B^	2.61^A^	31.38^B^	8.14^B^	64.43^B^
	WJEF46	4.18^B^	2.04^C^	32.61^B^	8.73^A^	60.95^C^
	WJEF63	4.49^A^	2.77^A^	34.08^A^	8.96^A^	67.2^A^

The data are the average of two independent replicates with five iterations each. At *p* < 0.05, values with the same superscript letter did not differ significantly. RPAE, Root P acquisition efficiency; RAPA, Root acid phosphatase activity.

### Rhizosphere biological properties after inoculation

The following table depicts the effects of various PSB inoculations on the biological properties of rhizosphere soils. We note that the PSBs strains application can decrease soil pH. The minimum pH value was found with the WJEF63 strain in both Broad Bean and Pea plants, which was 5.09 and 5.13, respectively. In the P-available parameter, the WJEF63 inoculation increased P-Available by 23.11% for Broad Bean and 22.77% Pea compared to the control, which confirms that the application of PSB strains was able to increase P-available in the soil. On the other hand, The PSBs inoculation had a significant impact on the rhizosphere’s soil N content, ranging from 19.57 to 56.95% (Table 6). The control show the lowest N content was found, in contrast, the WJEF63 treatment show the highest one, which was not significantly different from the values of other PSBs treatments. A similar trend was also observed for soil EC, with maximum values occurring in the WJEF63 strain 0.63 and 0.68 dS m^−1^ in Broad Bean and Pea treatment, respectively. The variation of the treatments’ measured alkaline phosphatase activity (APA) ranged between 7.29 to 22.47 μg pNP g^−1^. However, PSBs inoculation significantly affected the APA in the rhizosphere soil. The highest APA was determined in WJEF63 which was statistically different from all treatments. β-glucosidase (GA) activity varied between 1.89 and 6.2 mg pNP g^−1^. The highest GA was observed with WJEF63 strains and it was different from the control treatment statistically. In addition, Basal soil respiration varied between 0.75 and 1.35 mg CO_2_ g^−1^ 24 h^−1^. The values in the control treatments, which refer to the production of carbon dioxide when soil organisms respire, were typically lower than those in the other treatments.

**Table 6:** Mean values for soil parameters as influenced by the applied PSB and RP in legumes experiments.

Legumes	Strains	*N* Total	P Available	OM	pH	EC	BSR	APA	GA
Broad Bean	Control	21.4^d^	7.43^e^	1.1^e^	7.54^a^	0.21^d^	0.75^f^	7.29^e^	2.1^f^
	WJEF15	42.5^c^	23.42^c^	1.32^d^	5.29^bc^	0.49^c^	1.04^e^	21.02^d^	4.36^e^
	WJEF26	41.67^c^	20.78^d^	1.49^c^	5.36^b^	0.46^c^	1.19^d^	21.17^c^	5.86^c^
	WJEF38	55.83^a^	31.82^a^	1.92^b^	5.23^c^	0.58^b^	1.35^a^	22.31^a^	6.01^b^
	WJEF46	50.34^b^	29.07^b^	2.06^a^	5.17^c^	0.61^a^	1.27^b^	22.08^b^	5.47^d^
	WJEF63	51.37^b^	32.14^a^	1.88^b^	5.09^d^	0.63^a^	1.21^c^	22.47^a^	6.2^a^
Pea	Control	19.57^E^	7.21^E^	1.08^D^	7.21^A^	0.22^E^	0.95^E^	7.62^E^	1.89^E^
	WJEF15	44.69^D^	28.36^C^	1.54^C^	5.79^B^	0.57^B^	1.02^D^	20.73^D^	5.73^C^
	WJEF26	49.63^C^	25.94^D^	1.83^B^	5.47^D^	0.41^D^	1.09^C^	21.22^C^	5.25^D^
	WJEF38	52.32^B^	30.59^B^	2.16^A^	5.33^E^	0.66^A^	1.04^C^	21.06^C^	5.94^A^
	WJEF46	51.83^B^	31.38^A^	1.77^BC^	5.62^C^	0.52^C^	1.17^B^	22.36^A^	5.22^D^
	WJEF63	56.95^A^	31.66^A^	1.91^B^	5.13^F^	0.68^A^	1.24^A^	21.96^B^	5.69^B^

Data represent the mean± of two independent replicates with five repetitions each. The data are the average of two independent replicates with five iterations each. At *p* < 0.05, values with the same superscript letter did not differ significantly. *N* total (mg.Kg^−1^), P available (mg.Kg^−1^), EC, electrical conductivity (dS m^−1^); OM, organic matter (%); BSR, basal soil respiration (mg CO_2_ g^−1^ 24 h^−1^); APA, alkaline phosphatase activity (μg pNP g^−1^); GA, β-Glucosidase activity (mg pNP g^−1^).

**Figure 5:**
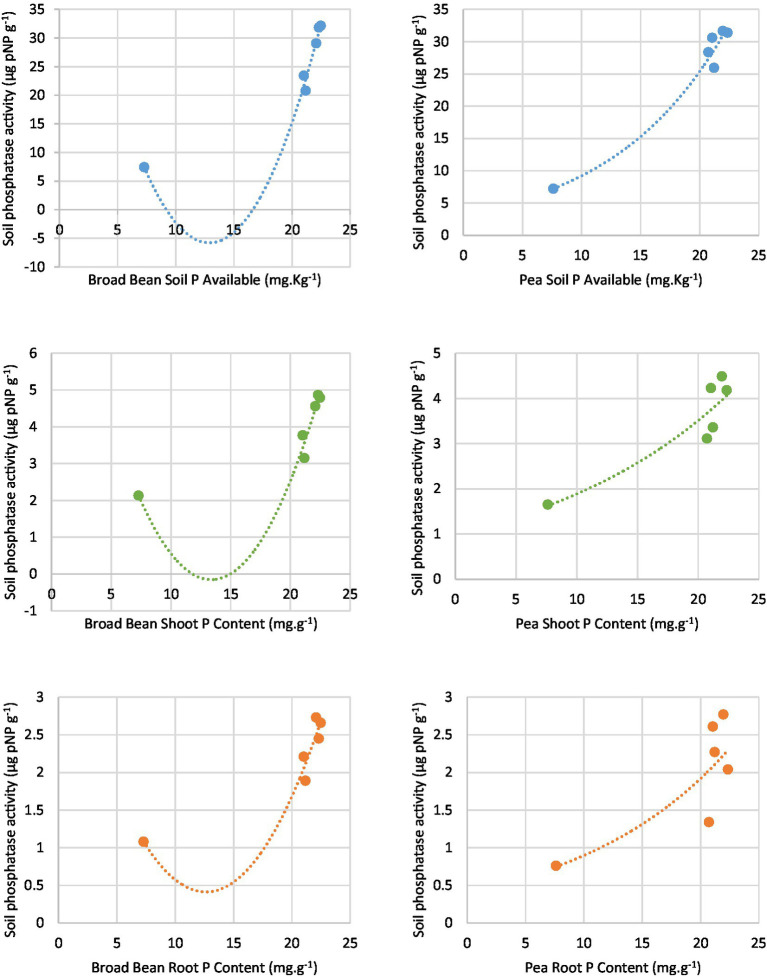
Correlation between rhizosphere phosphatase activity with soil available P, Soot P content, and Root P content.

According to the regression analysis (Table 7 and Figure 5), rhizosphere soil phosphatase activity (alkaline) was significantly associated with available P contents of soil, P contents in the shoot, and P contents in the root of both Broad Bean and Pea seeds. A polynomial model and exponential model resulting from the regression analyses of soil phosphatase activity and the other parameters.

**Table 7:** Mathematical modelling of P parameters in response to soil phosphatase activity.

P-parameters	Equation	Mathematical model	*R*^2^*
Broad Bean Soil P Available	*y* = 0.418*x*^2^–10.795*x* + 63.916	Polynomial	0.9832
Broad Bean Shoot P Content	*y* = 0.0614*x*^2^–1.6448*x* + 10.856	Polynomial	0.9437
Broad Bean Root P Content	*y* = 0.0234*x*^2^–0,5,902*x* + 4.141	Polynomial	0.9204
Pea Soil P Available	*y* = 3.3392e^0.1015x^	Exponential	0.9886
Pea Shoot P Content	*y* = 1.014e^0.0621x^	Exponential	0.8951
Pea Root P Content	*y* = 0.4199e^0.076x^	Exponential	0.7624

*R*^2^, weighting coefficient.

## Discussion

The majority of studies that were used to evaluate the effects of PSB inoculation were conducted in carefully monitored environments. The bacteria that perform the best in these studies are assessed in the field. The type of soil, the amount of nutrients present, and the species of the host plant are some variables that can influence how bacteria behave in soil. Field tests were conducted to assess different PSB strains that might help in the growth of a variety of crops, including chickpeas, wheat, corn, and barley (Masdariah Sembiring et al., 2019; Elhaissoufi et al., 2020). However, few studies have been conducted to evaluate PSB inoculation in Broad Beans and Peas. The current study raises the level of knowledge on the PSB-legumes interaction under insoluble P, specifically variations in root growth, as well as the associated rhizosphere modifications and above-ground growth related to P-use efficiency were studied using Broad Bean and Pea crops as tested legumes.

Soil bacteria are an important component of the agroecosystem playing an important role in soil nutrient cycling, pesticide degradation, soil health maintenance, and crop productivity (Meetei et al., 2022; Tarafdar, 2022). By increasing crop nutrient uptake and facilitating the efficient recycling of poorly soluble P in the soil, inoculating PSB strains increases the availability of poorly soluble P in the soil, which can reduce the need for P fertilizer application (Bargaz et al., 2021; Ajibade et al., 2023). In our study, a total of 51 PSBs were isolated from the rhizosphere soil samples under legume cropping systems at four experimental bases Tafrant, Ait Yaakoub, Dayt Al Amira, and Ait Saleh. Among them, five strains that had different RP-solubilizing capacities, with values ranging from 53.75 to 76.58 mg.L^−1^, were chosen as inoculum. These findings are consistent with those of Elhaissoufi et al. (2020) who determined the soluble RP range between 6.41 and 66.02 μg mL^−1^.

Additionally, PSBs’ numerous positive effects are frequently cited as important elements that encourage plant growth and raise soil P availability. The production of indole3-acetic acids (IAA), minocyclopropane1-carboxylate (ACC) deaminase, siderophores, ammonia, and P solubilization by these bacteria of various genera have been reported to promote plant growth (Sagar et al., 2020; Kapadia et al., 2021). According to studies, IAA can help crops grow and develop as well as absorb and use nutrients by encouraging the growth of root hairs and root elongation (Harahap et al., 2022; Song et al., 2022). The majority of the microorganisms isolated from the rhizosphere of a variety of crops can synthesize and release auxins as secondary metabolites (Gusain and Bhandari, 2019). All tested strains produce IAA except for WJEF26, the amount of IAA secreted by the WJEF38 strain was as high as 91.03 μg mL^−1^. Iron is one of the essential nutrients that, along with IAA, can restrict biotic metabolism and growth. By making available iron to host plants or by reducing available iron to pathogens in the environment, rhizospheric bacteria that produce siderophores may decrease pathogen competitiveness, promoting the growth, and development of plants (Elnahal et al., 2022). Siderophores are low-molecular-weight compounds that can bind Fe^3+^ and deliver nutrients directly to microbial cells. All isolated strains produced siderophores in this study except for WJEF26, with the activity of the four PSB strains ranging from 39.46 to 73.26 units. Tested strains were characterized by other bacterial traits such as HCN and ammonium-producing strains (equivalent to 98.37 μg mL^−1^) and were likely involved in non-symbiotic N_2_ fixation during plant growth. According to several studies (Elhaissoufi et al., 2020; Khoshru et al., 2020; Mahmud et al., 2021) ammonia production, medium acidification, and HCN production may all help seedling robustness, including crucial early-stage root nutrient absorptive capacity that could enhance RP solubilization and subsequent utilization.

Root germination and growth are known to be significantly influenced by PGP traits such as IAA, siderophores, and ACCD secreted by rhizosphere bacteria (Kapadia et al., 2022). Therefore, the possibility of treating seeds with the fermentation broth of particular strains was explored. It is evident from the seed germination experiment that PSBs helped Broad Bean and Pea seeds grow. The findings showed that a strong capacity for IAA production in conjunction with a high capacity for P solubilization can increase the percentage of seeds that germinate, significantly influencing the regulation of endogenous IAA levels with favourable effects on P uptake and plant physiological status. On their part, Yañez-Yazlle et al. (2021) SA211 is a significant producer of IAA, which primarily affects rooting, the growth of secondary roots, and the development of root hairs. Excess production of this auxin may be the root of the anomalous growth. In our study, which was based on the legumes pot experiment, higher acid phosphatase activities were found in the rhizosphere soil of Broad Bean and Pea roots that had been inoculated with PSBs. This may have the effect of accelerating the decomposition of organic P and inorganic P that is not yet available, which would further encourage legume plants to absorb P. Similar to this, Song et al. (2022) found higher acid phosphatase activities in the rhizosphere soil of maize roots inoculated with PSBs, which may hasten the decomposition of organic P and unavailable inorganic P, further promoting P uptake by maize plants. The P-solubilizing, IAA-synthesizing, acid phosphatase activity, and root colonization properties of the PSB strains may be responsible for the significant (*p* ≤ 0.05) increase in the tested legumes’ growth parameters, including plant P contents, following inoculation (Tables 4, 5). Since RP was used as an insoluble P source in the pot experiment, there may be a strong correlation between the rise in legume-P contents and the solubilization of RP. Similarly, Elhaissoufi et al. (2020) pointed out that the development of wheat seedlings may be related to the multiple properties of PGPs (i.e., IAA, siderophore, NH4+, etc.), which likely contribute to a further improvement in growth, in addition to the increased biosolubilization of P in the rhizosphere and changes in root morphology. Additionally, the fact that PSB strains have a relatively higher capacity to produce IAA may be the cause of their significant impact on root length and root fresh and dry weight. By increasing P-uptake from the soil and its transport to plant shoots, the inorganic P-solubilization and IAA production abilities of PGPR are known to enhance plant growth (Gupta and Buch, 2019; Wang et al., 2021). Following inoculation with PGPR strains, the promotion of root colonization and plant growth has also been previously discussed (Elhaissoufi et al., 2020; Khourchi et al., 2022). Under field circumstances, our study’s findings were incredibly inconsistent. When compared to non-inoculated plants, inoculated plants treated with RP fertilizer showed a significant (*p* ≤ 0.05) increase in plant height, N, and P contents. This has been approved by Khan H. et al. (2022) noted that soil inoculation of PSB with mineral P improved soil fertility post-harvest by increasing soil organic matter from 0.61 to 0.70%, lowering pH from 7.74 to 7.68, and increasing total soil nitrogen from 0.04 to 0.09%. The fact that sterilized soil was used in the pot experiment and, as a result, there was no competition between the inoculated bacteria and the native microflora, is one explanation for the effect of the PSB strains. Although RP-non-inoculation could not compete with RP-PSB-fertilizer treatments, significantly higher P contents were found in inoculated plants. This could be because the inoculated strains solubilized P.

The majority of earlier studies (Elhaissoufi et al., 2020; He and Wan, 2022) primarily discussed the direct effects of PSBs on plant growth based primarily on P solubilization and plant growth. Other studies have also documented significant positive effects on a small number of root parameters without a clear relationship to parameters related to aboveground P. This study, however, offered fresh proof that PSB effects extend beyond P-solubilization alone and include numerous known and unidentified indirect effects on the morphological shoot and root traits, enhancing both the acquisition and internal utilization of P. In this article, multiple differential responses at the level of PSB-plant interactions are reported. Of particular note is the significant rise in rhizosphere P availability because of PSB inoculation (Table 1), which may provide a sufficient amount of P for roots to grow, subsequently, better plant growth, and P nutrition, which is presumably guaranteed for later growth stages. Elhaissoufi et al. (2020) also show that P biosolubilisation by rhizosphere bacteria probably stimulates positive surface and subsoil interactions. These authors state that PSBs could play a key role in plant growth by promoting root development in 15 day-old seedlings more than P solubilisation, which appeared to be pronounced earlier on the seventh day after germination and tended to decrease in 15 day-old and 42 day-old wheat seedlings. In addition, we suggest that a successful growth and development phase after germination gives a strong impetus to the whole life of the plant. High germination accompanied by strong seedling cell development leads to high plant vigour, and vice versa. The small quantities of monomeric sugars released into the soil serve as a substrate for soil microorganisms, which move from a solitary to a symbiotic lifestyle. All this explains the importance of bacteria in the plant’s life cycle and development, and indirectly bacterial inoculations improve the plant’s biomass.

Because of the increased root absorption capacity and the potential internal remobilization of cellular P pools caused by increased root APase activity, PSB also tended to stimulate both root APase activity and RL (Elhaissoufi et al., 2020; Timofeeva et al., 2022). This is in line with earlier research that found PSB strains could produce APase to enhance P nutrition (Khourchi et al., 2022). Additionally, compared to non-inoculated plants, inoculated plants demonstrated a noticeable improvement in aboveground physiological traits, as evidenced by higher P and N levels. All of the treatments significantly increased the nitrogen and phosphorus contents in the above-ground parts of legume plants, and the results were consistent with those of earlier studies by Majeed et al. (2015, 2022). Because ammonium must be in a reduced state for the plant to use it, ammonia is preferable to nitrate or nitrite in the soil. The soil’s rhizoflora then transforms ammonia into a usable state. When incubated on N-containing media in the current study, nearly all of the isolates produced a significant amount of ammonia, suggesting that the isolated microbes were engaging in PGP activities to obtain and supply nitrogen to the plant rhizospheres and increase biomass (Emami et al., 2018). The multiple functions of PSBs also help plants better absorb nutrients, which increases the amount of nitrogen and phosphorus in above-ground plant parts. Better nutrient uptake by the plants is also a result of root growth in response to PSBs inoculation. Crop plants’ overall growth, development, and health are dependent on the availability of adequate nutrients, and they are controlled by their microbiome’s capacity to produce a range of traits that enhance plant growth. Multiple nutrients are provided to the plants through the inoculation of diverse rhizobacteria, aiding in plant growth (Gupta and Pandey, 2020). As shown in this study, two crops responded to PSBs by increasing their root and shoot biomass.

Different PSB properties that promote plant growth are associated with improved plant growth in legume plants. According to Gianfreda (2015), measurements of soil enzymes also indicate the health of the plants. In our findings, we note that all tested strains show secretion of ACCD ranging from 0.27 to 0.56 nmol α-ketobutyrate mg/protein/h. PGPR has many direct and indirect pathways for improving crop yields in stressed conditions, including the production of ACCD (Sagar et al., 2020). The enzyme ACCD reduces the level of ACC in root exudates; a low level of ACC reduces the concentration of ethylene in plant roots, which aids in root length and thus nutrient absorption (Sagar et al., 2022). The enzyme ACCD converts the ACC secreted by stressed plants into ammonia and-ketobutyrate, which has a significant impact on the physiology, growth, and development of the plant (Saleem et al., 2018). In such a study, *Aneurinibacillus aneurinilyticus* and P aenibacillus sp., which secrete ACCD, were inoculated in consortia and increased the weight and biomass of *Phaseolus vulgaris* seedlings under saline-stressed conditions (Gupta and Pandey, 2020). Soil enzyme activities and respiration provide information about the microbial population and their activities in the soil. Similar to the findings of this study. Gianfreda (2015) found that soil enzyme activity is higher in the rhizosphere when compared to bulk soil. Similar findings were made by Wang et al. (2023) who found that soils that had microbes added in addition to NPK had higher enzyme activities than sterile soils. According to Bargaz et al. (2021) the presence of insoluble P, for which PSB can produce enzymes and organic acids to solubilize this unavailable phosphorus, is what accounts for the higher alkaline phosphatase enzyme activity. Timofeeva et al. (2022) determined that the use of chemical fertilizers and PSBs activates soil enzymes at various levels. We determined that adding both chemical-and biological-fertilizers activates and boosts soil biological activity. Additionally, we can say that every strain originated from the rhizosphere, which explains their significant effect on the soil characteristics and, consequently, on the plant growth parameters. Agriculture and food production may benefit from the use of PSBs. Using these bacteria can lessen the need for P-fertilizers, which can be expensive and have negative environmental effects like polluting groundwater. Through the P-and nutrient cycle, they can also improve soil quality and raise crop yields. Future research is anticipated to strengthen the mathematical framework connecting the PSB’s potential and its contribution to the advancement of modern agriculture.

## Conclusion

Experiments on seed germination demonstrate that PSB strains can significantly encourage early plant growth while raising the germination rate of the two plants under study. Seeds treated with PSB had a higher germination rate and longer rooting length than the untreated controls. The pot experiment further demonstrated that PSB inoculation could enhance soil acid phosphatase activity and available P, as well as plant P uptake. Our research shows that PSBs from the rhizosphere soil of legumes can produce IAA, siderophores, HCN, and ammonia producers that support the growth of Broad Bean and Pea in addition to solubilizing P and secreting acid phosphatase to dissolve insoluble P. It is possible to conclude that these species have a high potential for reducing the use of chemical P fertilizers and promoting long-term agricultural development. Based on these findings, we conclude that PSB-tested strains are potential P-solubilizing and plant-growth-promoting strains that can supplement Broad Bean and Pea P requirements. Before using the five strains as commercial inoculants for improving Broad Bean and Pea crops, we recommended being thoroughly tested and identified. To understand the existence of microbial communities in different conditions and their effect in field conditions, a consortium of PGPs and their interactive effect must be studied at the molecular level. Further research into the mechanisms and experiments with these organisms in different plants would lead to the effective use of these PGPRs as bioinoculants for various soil properties. Many such studies and experiments conclude that PSBs strains are a viable alternative for plants to withstand abiotic stress and enhance plant growth promoters.

## Data availability statement

The original contributions presented in the study are included in the article/supplementary material, further inquiries can be directed to the corresponding author.

## Author contributions

All authors listed have made a substantial, direct, and intellectual contribution to the work and approved it for publication.

## Conflict of interest

The authors declare that the research was conducted in the absence of any commercial or financial relationships that could be construed as a potential conflict of interest.

## Publisher’s note

All claims expressed in this article are solely those of the authors and do not necessarily represent those of their affiliated organizations, or those of the publisher, the editors and the reviewers. Any product that may be evaluated in this article, or claim that may be made by its manufacturer, is not guaranteed or endorsed by the publisher.
